# Generalizability of machine learning methods in detecting adverse drug events from clinical narratives in electronic medical records

**DOI:** 10.3389/fphar.2023.1218679

**Published:** 2023-07-12

**Authors:** Md Muntasir Zitu, Shijun Zhang, Dwight H. Owen, Chienwei Chiang, Lang Li

**Affiliations:** ^1^ Department of Biomedical Informatics, College of Medicine, The Ohio State University, Columbus, OH, United States; ^2^ Department of Internal Medicine, College of Medicine, The Ohio State University, Columbus, OH, United States

**Keywords:** adverse drug events, electronic health records, machine learning, natural language processing, relation extraction

## Abstract

We assessed the generalizability of machine learning methods using natural language processing (NLP) techniques to detect adverse drug events (ADEs) from clinical narratives in electronic medical records (EMRs). We constructed a new corpus correlating drugs with adverse drug events using 1,394 clinical notes of 47 randomly selected patients who received immune checkpoint inhibitors (ICIs) from 2011 to 2018 at The Ohio State University James Cancer Hospital, annotating 189 drug-ADE relations in single sentences within the medical records. We also used data from Harvard’s publicly available 2018 National Clinical Challenge (n2c2), which includes 505 discharge summaries with annotations of 1,355 single-sentence drug-ADE relations. We applied classical machine learning (support vector machine (SVM)), deep learning (convolutional neural network (CNN) and bidirectional long short-term memory (BiLSTM)), and state-of-the-art transformer-based (bidirectional encoder representations from transformers (BERT) and ClinicalBERT) methods trained and tested in the two different corpora and compared performance among them to detect drug–ADE relationships. ClinicalBERT detected drug–ADE relationships better than the other methods when trained using our dataset and tested in n2c2 (ClinicalBERT F-score, 0.78; other methods, *F*-scores, 0.61–0.73) and when trained using the n2c2 dataset and tested in ours (ClinicalBERT F-score, 0.74; other methods, *F*-scores, 0.55–0.72). Comparison among several machine learning methods demonstrated the superior performance and, therefore, the greatest generalizability of findings of ClinicalBERT for the detection of drug–ADE relations from clinical narratives in electronic medical records**.**

## 1 Introduction

Adverse drug events (ADEs) are unintended harmful effects of taking medication (Hohl et al., 2018), which is a leading cause of death in the United States (Classen et al., 1997; Binkheder et al., 2022) and responsible for the hospitalization of 9,440,757 patients from 2008 to 2011, with an increasing trend over time (Poudel et al., 2017). The estimated annual cost of drug-related morbidity and mortality resulting from non-optimized medication therapy was $528.4 billion, equivalent to 16% of total US healthcare expenditures, in 2016 (Watanabe et al., 2018). Patients with ADEs have demonstrated significantly longer hospital stays and an almost two-fold greater risk of death than patients without ADEs (Classen et al., 1997). Nevertheless, ADEs are mostly preventable (Rommers et al., 2007), and early detection can substantially reduce morbidity and, thereby, decrease associated healthcare costs (Classen et al., 1997; Kaushal et al., 2006; Handler et al., 2007).

ADEs are largely detected after marketing, so timely surveillance at this time is important for patient safety (Botsis et al., 2011; Polepalli Ramesh et al., 2014). Pharmacovigilance has traditionally employed spontaneous reporting systems (SRSs), but as many as 90% of ADEs may remain unreported in this voluntary scheme (Hazell and Shakir, 2006). In contrast, electronic health records (EHR) represent a potentially great source for post-marketing surveillance of drug safety, accommodating real-time clinical data gathered from routine clinical care (Coloma et al., 2013). One study revealed relevant ADE information, for example, in the structured data of 9,020 of 31,531 patients (28.6%) with side effects of statin documented in provider notes (Skentzos et al., 2011). Furthermore, clinical notes in EHRs provide longitudinal information related to drug-induced adverse events, but the manual review and extraction of ADEs from enormous clinical narratives is labor intensive, and clinical notes in EHRs vary from patient to patient, physician to physician, and hospital to hospital. Therefore, an automated system that utilizes artificial intelligence (AI) is needed to extract ADEs from clinical notes, and attempts have been made to build such a system.

The Medication and Adverse Drug Events Challenge (MADE1.0) (Jagannatha et al., 2019) aimed to automatically identify clinical concepts and relations from clinical narratives that included ADEs. The Challenge included three tasks: 1) naming the recognized entity (NER) and identifying the medication and its route, dosage, duration, frequency, and indication, as well as associated ADEs and their severity; 2) identifying relations (RI) of medications with ADEs, indications, and other entities; and 3) performing the NER and RI tasks jointly. The Challenge released 1,089 fully de-identified clinical notes from 21 randomly selected patients with cancer at the University of Massachusetts Memorial Hospital that included 2,612 drug–ADE relations. Methods used to classify relations ranged from statistical machine learning (ML)-based methods, such as support vector machine learning (SVM), random forest, and others, to neural-network-based bidirectional long short-term memory (BiLSTM). The best-performing model for the classification of ADE–drug name relations achieved an *F*-score of 0.72.

Another effort, the n2c2 Shared Task Challenge (Henry et al., 2020), mirrored MADE1.0 and included similar tasks. The n2c2 dataset comprised 505 discharge summaries taken from the Medical Information Mart for Intensive Care-III (MIMIC-III) clinical care database (Johnson et al., 2016). Records were selected by searching ADEs in the International Classification of Diseases (ICD) code descriptions of the records, which yielded a total of 1,840 ADE-drug relations. Methods used for the relation-classification task ranged from SVM to attention-based BiLSTM, with the best-performing model for ADE-drug name relation yielding an *F*-score of 0.85.

The use of data from a single EHR source in the two challenges allowed NLP approaches developed from these models to be data-specific. The model that performed the best (Wei et al., 2020) in relation extraction for the n2c2 Shared Task, for example, contained a post-processing technique that was dependent on the n2c2 annotation guideline to improve the performance of their BiLSTM conditional random field (CRF) algorithm. This n2c2 paper (Wei et al., 2020) also suggested that language models like BERT (Devlin et al., 2018) in the biomedical domain may further improve the NLP performance, and it remains to be an interesting topic for future research. In the best-performing model (Chapman et al., 2019) from the MADE 1.0 Challenge, the best model was random forest. The paper (Chapman et al., 2019) stated that generalizability of the best-performing model was unclear.

Though transformer-based methods like BERT (Devlin et al., 2018), BioBERT (Lee et al., 2020), and ClinicalBERT (Alsentzer et al., 2019) have become popular in recent years, they have not been applied to identify ADEs from the clinical notes of EHRs. Therefore, it is critical to implement the same model on data from different data sources to assess the generalizability of a model. No study has been conducted yet that used EHRs from different data sources to address the issue of generalizability. BERT-based methods have performed better than other machine and deep learning methods (Sun et al., 2019; González-Carvajal and Garrido-Merchán, 2020; Minaee et al., 2022) in mining biomedical texts, and methods that integrate biomedical corpora, such as ClinicalBERT, outperformed BERT models (Huang et al., 2019). In this article, we investigate the performance of deep learning (CNN, BiLSTM) and transformer-based (BERT and ClinicalBERT) methods, as well as classical SVM, in clinical notes of two different EHR systems.

The preprocessing of data is important in NLP in clinical text and challenged by the inherent variations in EHRs, and the preprocessing of clinical text in EHRs, such as tokenization, which breaks the text into meaningful elements and detects sentence boundaries, is different and more challenging than the processing of data in the literature (Griffis et al., 2016). Publications often inadequately present the end-to-end data preprocessing method, but in this paper, we sufficiently detail the end-to-end data preprocessing for all NLP methods used.

Our primary goal is to address whether and how ADE NLP algorithms developed from the n2c2 Shared Task can be generalized to a drug-specific ADE in a different EHR. There are two types of generalizability in the NLP algorithm development for detecting drug-induced ADEs. First, we want to know whether the NLP model developed in one dataset maintains a comparable performance in a different dataset. Second, if one NLP method has a better performance than the others, will it maintain its supreme performance in a different dataset? In this paper, we study both types of generalizability for NLP algorithms designed for drug–ADE relationship detection. We will use ADEs induced by immune checkpoint inhibitors (ICI) (Nashed et al., 2021) as recorded in clinical notes as examples for analysis. MADE1.0 (Jagannatha et al., 2019) data were not available to us when we conducted this study.

## 2 Materials and methods

### 2.1 Datasets

We used two datasets to analyze the generalizability of ADE detection from EHRs; one was developed from the EHR system of The Ohio State University James Cancer Hospital, and the second was that of the n2c2 Shared Task (Henry et al., 2020).

### 2.2 ICI-OSU corpus

**FIGURE 1:**
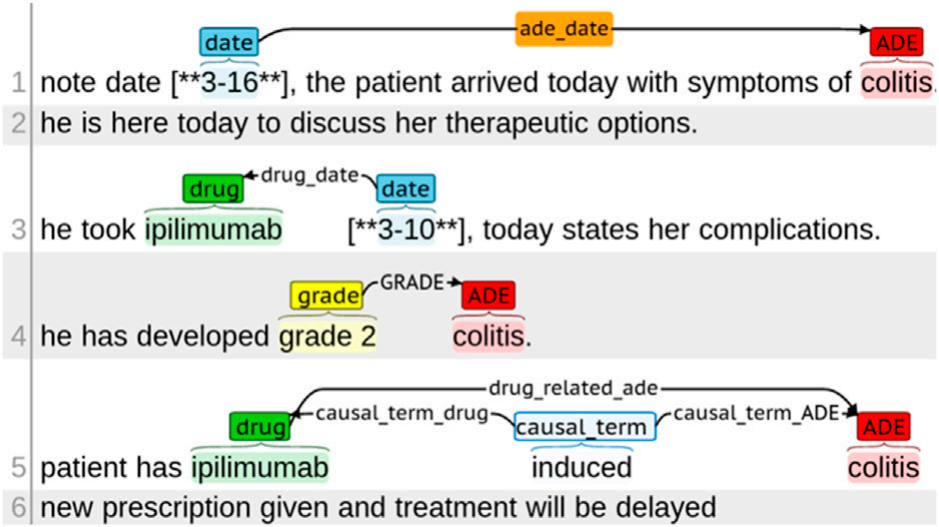
Annotation example for different entities in the ICI-OSU corpus.

We built the ICI-OSU corpus by manually annotating 1,394 clinical notes of 47 randomly selected patients who received immune checkpoint inhibitors (ICIs) from 2011 to 2018 at The Ohio State University James Cancer Hospital. Supplementary materials contain a detailed annotation guideline that we developed to assist the annotators with manual annotation. Supplementary Table S1 contains the annotation guideline for entity annotation, whereas Supplementary Table S2 contains the guideline for relation annotation. Figure 1 shows different types of entity tags and relation annotations.

The entities included drug names, mentions of ADEs, dates drugs were taken, dates of ADEs, terms drawing causal relation between a drug and an ADE, and grades of ADE intensity. It is worth mentioning that *reasons for drug administration* were not considered as ADEs. Supplementary Figure S1 shows the difference between ADE and *reason for drug administration* with an example. Relations were annotated for entities located within a single sentence and across sentences. Two annotators with informatics skills and knowledge in cancer clinical trials independently annotated each note, and a third annotator performed the validation for inconsistent annotations between the two annotators. One annotator was a resident physician with hands-on experience with EHRs; the second had a master’s degree in biology with 7 years of experience in corpus development and annotation; the third annotator, who performed the validation, was a graduate student in biomedical informatics. The institutional review board of The Ohio State University approved this study (#2020C0145).

### 2.3 ICI-OSU data processing pipeline

**FIGURE 2:**
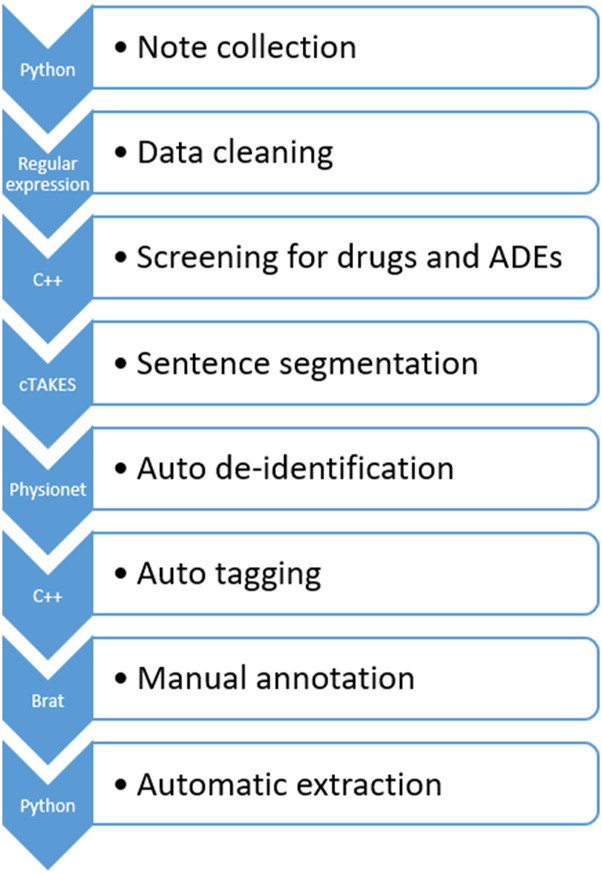
ICI-OSU data processing pipeline. Python and C++ programming were used at different steps to automate the process.

Figure 2 illustrates the data processing pipeline we built that feeds processed data to the ADE NLP models for generalizability analysis.

In the first step, we collected the clinical notes of the targeted patient. The ICI-OSU corpus contains the patient’s notes for the first 12 months from the date of the first ICI dose in the structured data. The order of note dates is maintained to keep track of longitudinal data, such as the date of first drug use, date of first ADE, and date of drug discontinuation.

In the second step, we cleaned data using regular expression techniques (Wang et al., 2019), which included but were not limited to normalizing uneven spaces and drug names and manipulating abbreviations. For drug name normalization, we used the DrugBank database (Wishart et al., 2008) as our primary source, and we built a lookup table that contained all possible mentions (such as the generic and brand name) of a drug. Supplementary Figure S2 shows a raw original clinical note, and Supplementary Figure S3 shows the cleaned data.

In the third step, we performed automatic screening for drugs and ADEs, tagging predefined drug and ADE terms for follow-up manual annotation. Table 1 delineates the drug names and the ADE list of our study. We used the Common Terminology Criteria for Adverse Events (CTCAE) (Freites-Martinez et al., 2021) and the DrugBank database as our primary guidelines for ADE and drug mentions, respectively. Then, we built a lookup table for screening that contained all possible mentions of these drugs and ADEs found in EHRs. We discussed with a physician and annotators who had hands-on experience working with EHRs, thus enriching and verifying our lookup table to ensure accuracy.

**TABLE 1:** List of drug names and adverse drug events. We used all possible mentions of these drugs and ADEs in our study.

Drug	ADE
Atezolizumab	Colitis
Ipilimumab	Pneumonitis
Nivolumab	Thyroid
Pembrolizumab	Abnormalities
Tremelimumab	Rash/dermatitis
Avelumab	Hepatitis
Durvalumab	Myalgia/arthralgia
Cemiplimab	Cardiotoxicity

In the fourth step, we used the Apache clinical Text Analysis and Knowledge Extraction System (cTAKES)™ (Savova et al., 2010), an open-source NLP tool, to segment sentences in the EHRs, which is one of the most challenging tasks because of variations, such as in use of punctuation and abbreviations, that are unique to the recorder (Griffis et al., 2016). To optimize cTAKES™ performance on sentence segmentation, we also encrypted the unexpected line breakers to be consistent with the cTAKES™ segmentation rules. After segmentation, we decrypted the data to preserve their originality. Supplementary Figure S4 shows the data’s appearance after sentence segmentation in cTAKES™.

In the fifth step, we performed automatic de-identification using de-identification software (Neamatullah et al., 2008) from PhysioNet (Goldberger et al., 2000) followed by manual de-identification by the annotators to ensure accuracy. The recall value of PhysioNet was over 98% in the de-identification task on our dataset. Supplementary Figure S5 shows the data after de-identification.

In the sixth step, we performed automatic tagging, annotating the drug name, mention of ADE, and date of ADE, to reduce the burden of subsequent manual annotation. For drug and ADE annotation, we performed exact matching of the data with our predefined drug and ADE lists. We used a rule-based method to annotate the dates in the clinical text automatically, and this annotation was consistent with that of the web-based brat rapid annotation tool (BRAT) (Stenetorp et al., 2012), which we used later for manual annotation. Though automatic annotation greatly reduced the burden of manual annotation, the annotators were free to annotate any new form of a drug name, mention of ADE, or date of ADE. The annotators could also correct anything incorrectly tagged in automatic tagging. Supplementary Figure S6 shows the automatically annotated notes.

In the seventh step, our annotators performed manual annotation in BRAT following the well-defined guideline mentioned previously and included in the supplementary materials. Supplementary Figure S7 shows the manually annotated notes.

In the eighth and final step, we extracted relevant annotation information automatically after manual annotation to build our corpus. Information extracted to prepare the data for machine learning models included drug–ADE relations, drug–ADE pairs, and neighboring words. We built an automatic system to extract that information from the annotated corpus based on the input format of the ML models. Due to the repetitive nature of EHRs, several drug–ADE relations were repeated when the text between a drug and an ADE, as well as the context, were exactly the same. We removed those duplicates in the automatic extraction.

### 2.4 n2c2 Shared Task corpus

The n2c2 data consisted of information from 505 discharge summaries taken originally from the MIMIC-III clinical care database (Johnson et al., 2016). The data provider described their process as first searching for ADEs in the ICD code description of each record and then manually screening the records with at least one ADE and dividing the data into a training set comprising 303 annotated files and a testing set that included 202 files (Henry et al., 2020). The n2c2 data contained several clinical concepts and relations as well as drug and ADE annotations, and we performed preprocessing as described previously to prepare the data for the ML models, first cleaning the n2c2 data, then segmenting sentences using cTAKES™, and finally using our automatic system to extract relevant annotations.

### 2.5 The definition of positive and negative drug–ADE relations in the n2c2 and ICI-OSU corpora

Like other researchers (Wei et al., 2020), we considered all possible combinations of drugs and ADEs to build positive and negative data for training and validating NLP models. Our generalizability analysis focused on drug–ADE relations within a single sentence; so, for example, for a sentence containing the drugs *d1* and *d2* and the ADEs *a1* and *a2,* the four possible drug–ADE combinations are (*d1*, *a1*), (*d1*, *a2*), (*d2*, *a1*), and (*d2*, *a2*). A drug–ADE relation was considered positive if the drug induced the ADE. We collected the positive samples directly from annotation to build the positive dataset. A relation was considered negative if the drug did not induce the ADE and was, therefore, not annotated in the corpus. We derived the negative dataset from all the drug–ADE combinations by subtracting the annotated positive set from the corpus. After removing duplicates, we obtained 189 positive samples and 698 negative samples from our annotated ICI-OSU data. The default n2c2 training and test data yielded 1,355 positive and 865 negative samples after duplicates were removed.

### 2.6 Machine learning deep learning models

We implemented several machine learning, deep learning, and transformer-based models, including SVM (Joachims, 1998), CNN (Kim, 2014), BiLSTM (Sherstinsky, 2018; Xu et al., 2019), BERT (Devlin et al., 2018), and ClinicalBERT (Alsentzer et al., 2019), to analyze the n2c2 and ICI-OSU datasets, and we trained these models on one dataset and validated them on the other to analyze the generalizability of their findings.

#### 2.6.1 Training and validation data

Training and validation data were speculated under the intra- and inter-dataset settings. The intra-dataset setting comprised training and validation data allocated from the same data source, either n2c2 or ICI-OSU. When training and validating the model on the same n2c2 dataset in the intra-set setting, we used the default training and validation data of the n2c2 dataset given by the data providers. Because the ICU-OSU dataset was relatively smaller, we applied five-fold cross-validation to avoid overfitting when we trained and validated our model on the same ICU-OSU dataset in the intra-set setting. In the inter-set setting, training was performed on one dataset, and its validation was performed in the other. We also withheld 30 percent of the data from the training set to serve as the internal validation set in the inter-dataset setting.

#### 2.6.2 Hyperparameter selection and embedding

In the deep learning and transformer-based models, we studied different dropout rates ranging between 0.1 and 0.8 and learning rates (0.1, 0.01, 0.001, and 0.0001) with batch sizes of 16, 32, and 64. In those models, we used embedding (Mikolov et al., 2013) techniques and pre-trained word-embedding models, such as Stanford’s global vectors for word representation (GloVe) (300-dimensional) (Pennington et al., 2014). In our transformer-based model, we also incorporated biomedical domain knowledge.

#### 2.6.3 Preparation of input data, feature selection, and model implementation

**FIGURE 3:**
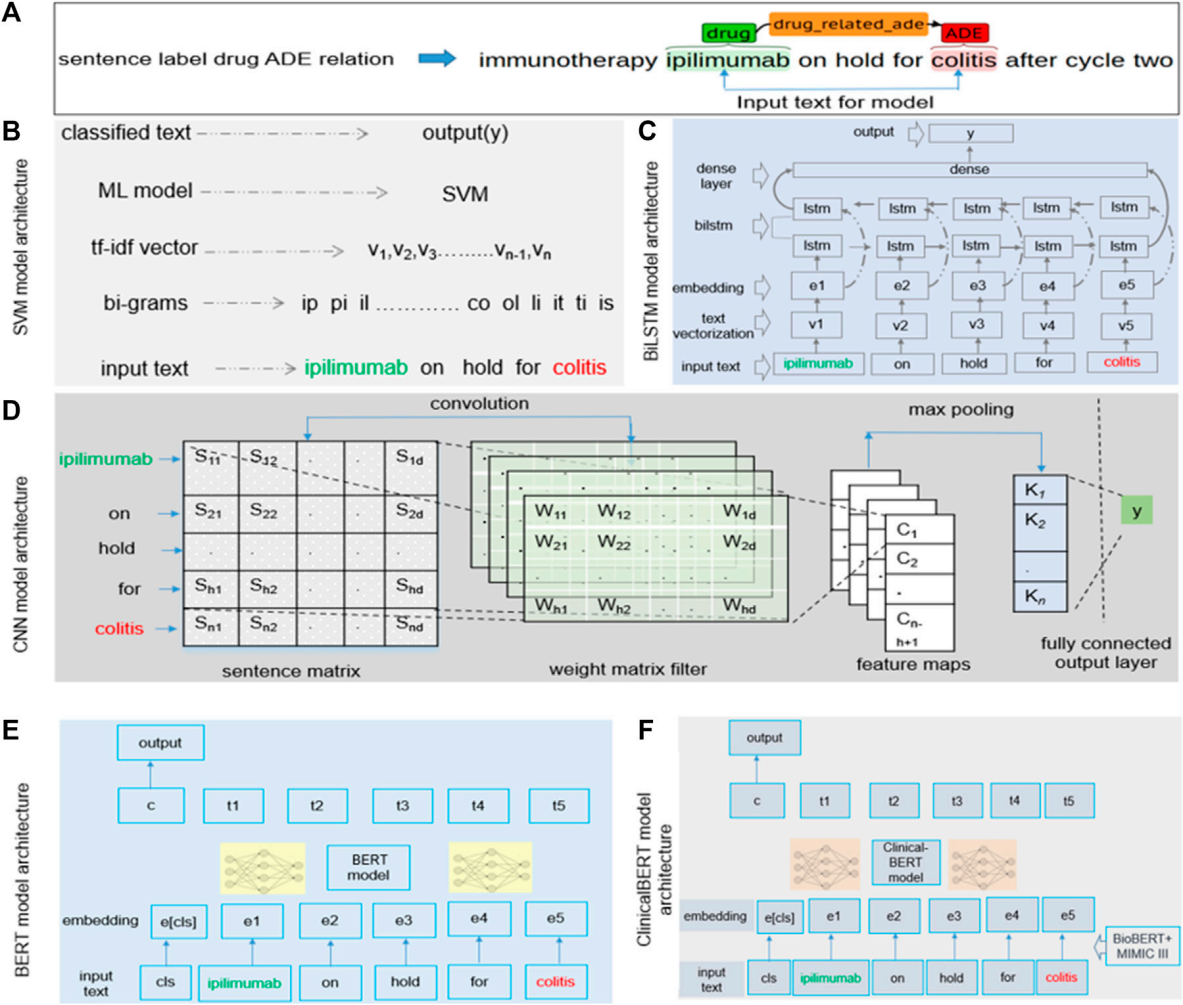
**(A)** Input text processing from single-sentence drug–ADE relation. **(B–F)** Implementation details of **(B)** SVM, **(C)** BiLSTM, **(D)** CNN, **(E)** BERT, and **(F)** clinicalBERT models.

We focused on the contextual features while building the models. Because the text between a drug and an ADE contains most of the contextual information regarding a drug–ADE relation, we considered the drug name, the ADE, and the text between them as the input for all models. However, feature selection varied from model to model. Our SVM model, for example, used character-level *n*-grams, whereas the CNN model considered *n*-grams at the token level. Figure 3A shows how we extracted the input text from a drug–ADE relation.

### 2.7 Implementation of the support vector machine model

We generated character *n*-grams from the first character of a sample to the last character of the input text using a range of values for *n*, converted those *n*-grams in term frequency (TF)-inverse document frequency (IDF) (Qaiser and Ali, 2018) vectorization, and then finally fed the feature vector into the model to predict the output *y* (0 or 1). We searched a range of values for *c* and gamma to obtain the best hyperparameter set and used the radial basis function (RBF) kernel in our SVM model. Figure 3B details the implementation of our SVM model with an example bigram.

### 2.8 Bidirectional long short-term memory (BiLSTM)

Understanding the context of a sentence is critical and requires that classification of the sentence includes information in both directions, from the beginning of the sentence to its end and from its end to its beginning. Previous studies showed promising results using BiLSTM to extract contextual information (Xu et al., 2019). BiLSTM algorithms can learn long-term dependencies and work in both directions of text and learn contextual features in a given time stamp. For our model, the input was a sequence from the start to the end of an entity of a sample. Figure 3C shows the architecture of the BiLSTM model. We performed text vectorization on the input and then used the pre-trained word embedding of GloVe (300d) (Pennington et al., 2014) in the embedding layer. The BiLSTM layer was used over the embedding layer, and finally, the dense layer was used, producing the output, *y*.

### 2.9 Convolutional neural network (CNN)

We implemented the standard CNN model (Kim, 2014), in which we focused on contextual information while extracting features. The CNN model applies a filter to extract features from text and uses those features to classify the text. As mentioned earlier, we used the text between the start of an entity and its end to analyze sequential words to learn features to extract. We used multiple filters of different sizes (2, 3, and 4) to examine different *n*-grams within the text. Figure 3D shows the architecture of the CNN model. We built a sentence matrix, with rows indicating the tokens of a sentence and columns indicating the features in which we implemented pre-trained word embedding. *S*_*n*_ is the number of tokens in a sentence; *S*_*d*_ is the feature dimension of a token; *W*_*h*_ is the kernel size. We then applied weight filters for the convolution operation and feature mapping and, finally, applied max pooling and fully connected the output layer to generate output. We used the rectified linear unit (ReLU) activation function and the pre-trained word embedding of GloVe (300d) in the embedding layer (Pennington et al., 2014).

### 2.10 Bidirectional encoder representations from transformers (BERT)

The BERT (Lee et al., 2020) model is based on a transformer encoder that uses a self-attention mechanism for sequence modeling (Vaswani et al., 2017). For our classification task, the sequential information of the text could be important. We selected a segment from the start to the end of an entity of a sample and then used the tokenized segment as input in the BERT model. The class token *cls* was added with the input. To obtain embeddings for our text, we used the pre-trained transformer model rather than an embedding layer. For our analysis, we used BERT-base, which consists of transformer blocks of 12 layers with 12 self-attention heads, a hidden size of 768, and 110 M trainable parameters (Lee et al., 2020). Figure 3E shows the architecture of the BERT model.

### 2.11 ClinicalBERT

The implementation of the ClinicalBERT (Alsentzer et al., 2019) model was similar to that of our BERT model, but ClinicalBERT incorporated pre-trained biomedical domain knowledge. We used the model that was initialized on BioBERT (Lee et al., 2020) and trained on all notes of the MIMIC-III dataset (Johnson et al., 2016). The model had a batch size of 32 and a maximum sequence length of 128 (Alsentzer et al., 2019). Figure 3F shows the architecture of the ClinicalBERT model.

## 3 Results

Annotation performance: we performed annotation in two rounds. In round one, we annotated 118 single-sentence positive drug–ADE relations and 24 cross-sentence positive drug–ADE relations. In round two, we labeled 163 single-sentence positive drug–ADE relations and 27 cross-sentence positive drug–ADE relations. Table 2 shows the average inter-annotator agreement (IAA) results of our two rounds of annotations. We calculated Cohen’s kappa (McHugh, 2012) to measure IAA. The results indicate considerable disagreement between the two annotators regarding the identification of drug–ADE relations, which is probably attributable to the diverse nature of ADE mentions in clinical notes. Supplementary Table S3 shows more annotation results and findings details of our corpus. Our OSU-ICI corpus is the first drug class-specific drug–ADE corpus. By specifically targeting ICIs, it also becomes a golden standard for developing immunotherapy-induced adverse event phenotypes.

**TABLE 2:** Agreement between annotators.

Type	*F*-score
Drug	99.00%
ADE	95.12%
Grade	70.66%
Causal term	73.58
Drug–ADE	70.94

### 3.1 Performance evaluation and error analysis

**FIGURE 4:**
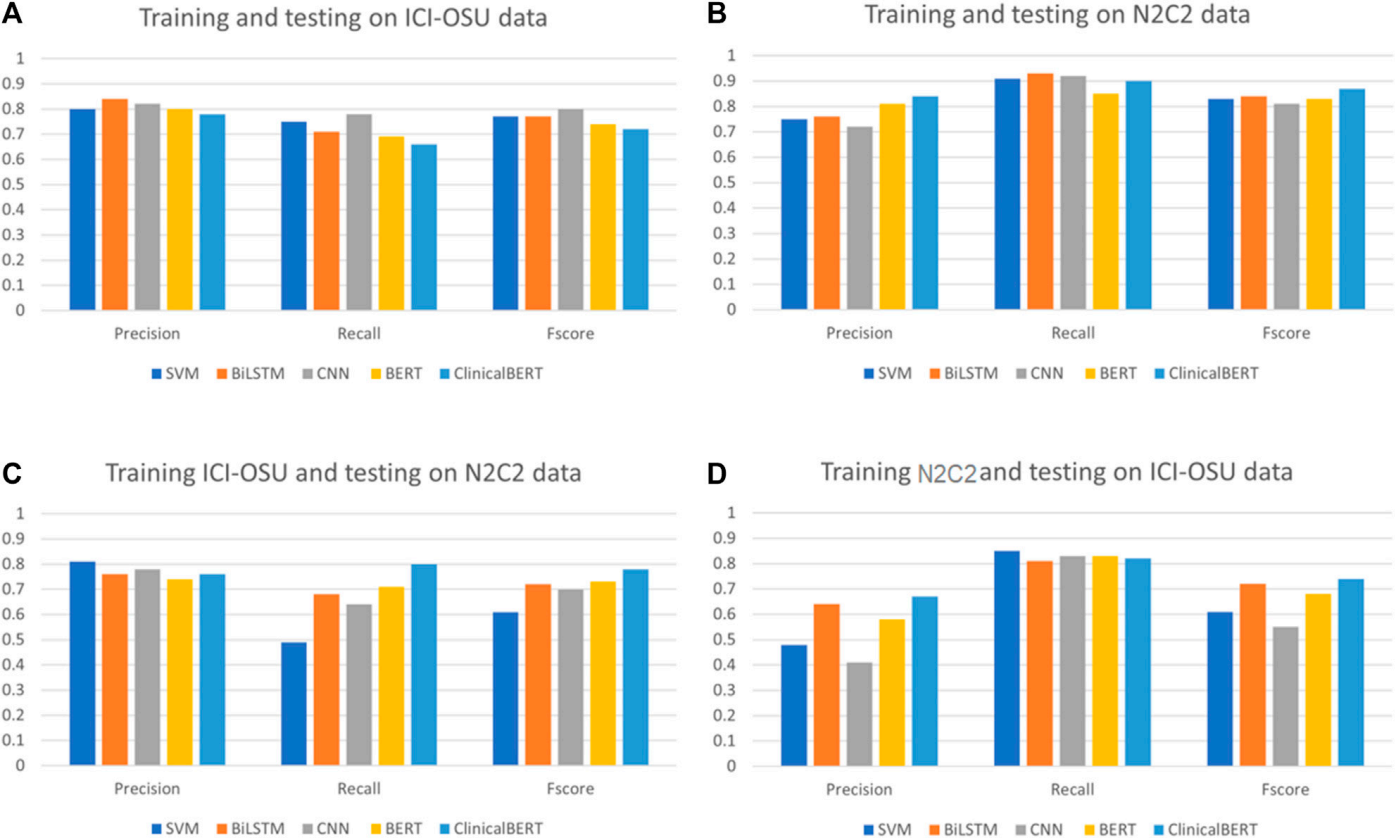
Comparison of precision, recall, and *F*-score among different models in intra- and inter-dataset settings. **(A, B)** show the performance of the different machine learning models when trained and tested on the same data source (intra dataset). **(C, D)** show the performance of the different machine learning models when trained and tested on different data source (inter dataset).

**FIGURE 5:**
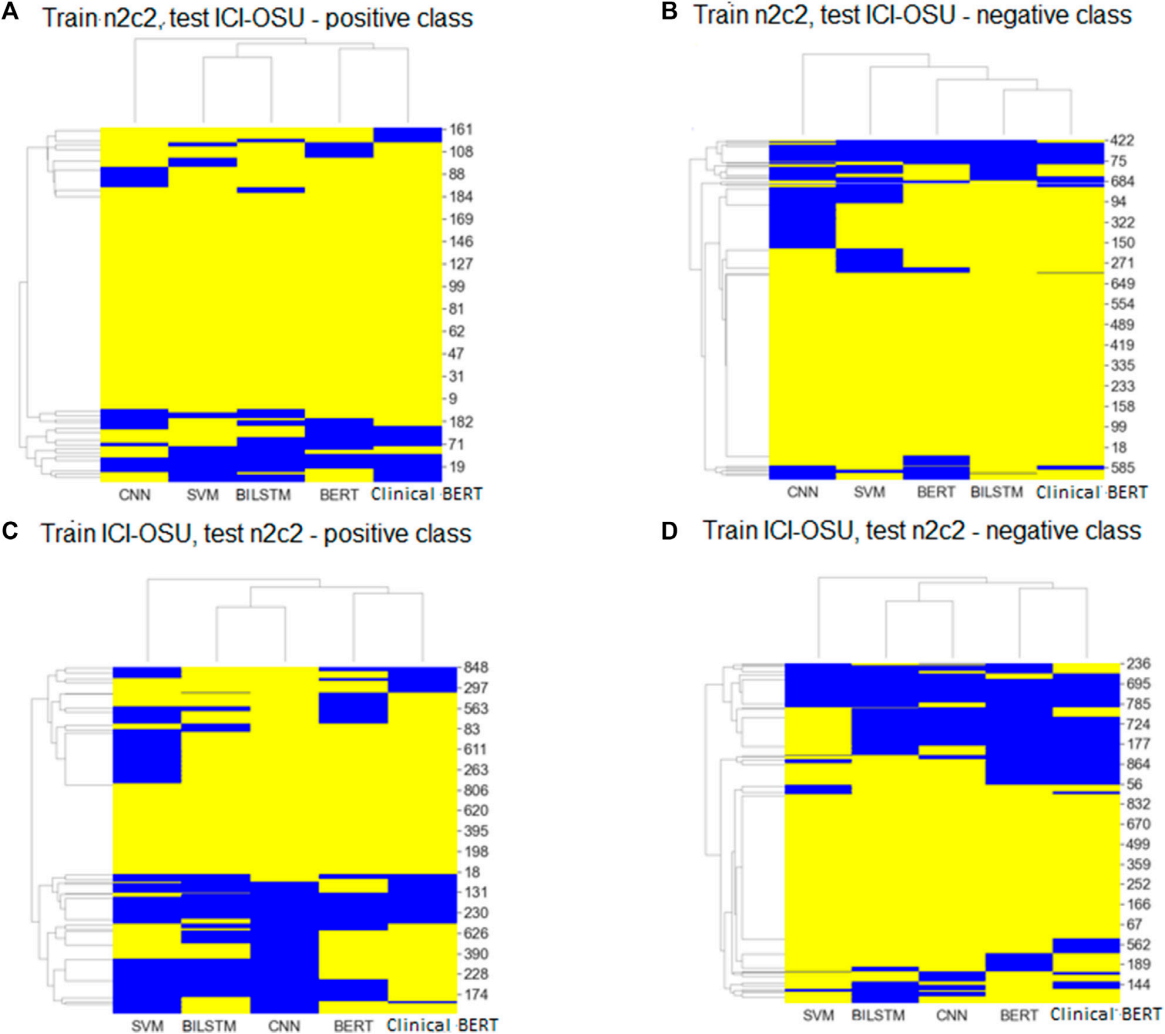
Cluster maps of results comparing predicted labels with true labels for every sample of positive and negative classes in inter dataset settings. Yellow: predicted and true label matched; blue: labels did not match. **(A, C)** shows the results for positive class. **(B, D)** shows the results for negative class.

To analyze the generalizability of findings among the models, we trained and tested each model on the dataset of the same data source (intra-data) and a different source (inter-data). Figures 4A, B show the performance of the different models in the intra-dataset setting. The CNN-based deep learning model performed the best, with an *F*-score of 80% for the ICI-OSU dataset, whereas the transformer-based ClinicalBERT model achieved the best *F*-score (87%) for the n2c2 data. The SVM model was also competitive and showed stable performance in the intra-dataset setting. Our results demonstrate that we did not get the best performance from either the BERT or ClinicalBERT model when training and testing on the ICI-OSU dataset. This is probably because the transformer models usually perform better on a large dataset, and the ICI-OSU dataset is small. Figures 4C, D show the performance of the different models in the inter-dataset setting, with the transformer-based models showing superior performance. The ClinicalBERT model achieved the highest F-score in both combinations of inter-data training and testing, probably because of the incorporation of domain knowledge as ClinicalBERT was trained on the MIMIC-III dataset. These performances indicate the importance of incorporating domain knowledge in models. Figure 5 shows the cluster map for the results of different models in the inter-dataset setting. In this NLP analysis of four combinations of training sets and test sets from n2c2 and ICI-OSU datasets, it is evident that ClinicalBERT has the best performance in three out of four combinations.

Figures 5A, B show the cluster map while training on the n2c2 dataset and testing on the ICI-OSU dataset. The BERT and ClinicalBERT models performed similarly for positive sample prediction, whereas the SVM and BiLSTM models clustered together with similar results. BiLSTM and ClinicalBERT models performed similarly for negative sample prediction. Figures 5C, D show the cluster map while training on the ICI-OSU dataset and testing on the n2c2 dataset. The BERT and ClinicalBERT models performed similarly for both positive and negative sample prediction, and the SVM and BiLSTM models clustered together with similar results. Comparison of the results of Figures 4 and 5 demonstrates the better performances of the machine learning and deep learning models in the intra-dataset setting and of the transformer-based models in the inter-dataset settings. Along with the internal capacity of the models to recognize patterns, the variable length of the datasets might contribute to such performance. Supplementary Tables S4 and S5 further detail the results.

### 3.2 Factors contributing to error 1 (differences in data sources)

The n2c2 dataset was the more general of the two sets and looked for all drug mentions in a clinical note, whereas the ICI-OSU dataset focused on ICI drugs and annotated a specific set of ADEs. The use of these differing types of data challenged training on one source and testing on the other.

### 3.3 Factors contributing to error 2 (differences in annotation guidelines and causal terms)

The annotation guidelines for the two datasets differed. The guideline for the n2c2 dataset, for example, looked for the closest entity rather than causation to draw a relation, whereas the guideline for the ICI-OSU dataset looked for the presence of causal terms to identify a causal relation between entities.

## 4 Discussion

### 4.1 Incorporation of domain knowledge

We attempted to analyze the generalizability of ADE detection from clinical notes using several machine learning, deep learning, and transformer-based models and observed promising performance, particularly when we applied state-of-the-art transformer-based models. The superior performance of ClinicalBERT indicated the importance of incorporating domain knowledge when using pre-trained data. Thus, future studies should incorporate more domain knowledge to further enhance the performance of the models.

### 4.2 Cross-sentence relation

Our study focused on single-sentence drug–ADE relations, in which the drug and ADE occurred in the same sentence, and after sentence segmentation and annotation, we ended with only a few cross-sentence relations. However, it is also important to identify cross-sentence relations. Our primary challenge in identifying cross-sentence relations was the imbalance within a dataset that could pose a very large number of negative relations against a very small number of positive relations. In addition, our experience in manual annotation showed that a drug and ADE could be distantly related across sentences with numerous sentences in between. Nevertheless, limiting the number of sentences between two entities to draw a relation could help limit the search space to accommodate most of the positive relations across sentences. It would also keep the number of negative relations considerably low.

### 4.3 Variation in sentence length

Variations in sentence lengths, some only a few words and some extraordinarily long, made it difficult to train the model and contributed greatly to the error. The ICI-OSU dataset included two positive samples consisting of 30 tokens each, where every single model except BERT classified them incorrectly. BERT was able to predict the true label of one of those two samples correctly. A lack of similar training data probably contributed to the error. Having more training data of similar length or building a separate rule-based approach could facilitate the management of extraordinarily long sentences.

## Data Availability

The datasets presented in this article are not readily available because the clinical notes of EHRs were used in this study. Therefore, data could not be published at this point. Requests to access the datasets should be directed to LL, Lang.Li@osumc.edu.

## References

[B1] Alsentzer E. Murphy J. R. Boag W. Weng W. Jin D. Naumann T. 2019. Publicly available ClinicalBERT embeddings

[B2] Binkheder S. Wu H-Y. Quinney S. K. Li L. Gao Y. Skaar T. C. (2022). PhenoDEF: A corpus for annotating sentences with information of phenotype definitions in biomedical literature. J. Biomed. Semant. 13 (1), 17. 10.1186/s13326-022-00272-6 PMC9188713 35690873

[B3] Botsis T. Nguyen M. D. Woo E. J. Markatou M. Ball R. (2011). Text mining for the vaccine adverse event reporting system: Medical text classification using informative feature selection. J. Am. Med. Inf. Assoc. 18 (5), 631–638. 10.1136/amiajnl-2010-000022 PMC3168300 21709163

[B4] Chapman A. B. Peterson K. S. Alba P. R. DuVall S. L. Patterson O. V. (2019). Detecting adverse drug events with rapidly trained classification models. Drug Saf. 42 (1), 147–156. 10.1007/s40264-018-0763-y 30649737 PMC6373386

[B5] Classen D. C. Pestotnik S. L. Evans R. S. Lloyd J. F. Burke J. P. (1997). Adverse drug events in hospitalized Patients&lt;subtitle&gt;Excess length of stay, extra costs, and attributable mortality</subtitle&gt;. JAMA 277 (4), 301–306. 10.1001/jama.1997.03540280039031 9002492

[B6] Coloma P. M. Trifirò G. Patadia V. Sturkenboom M. (2013). Postmarketing safety surveillance: Where does signal detection using electronic healthcare records fit into the big picture? Drug Saf. 36 (3), 183–197. 10.1007/s40264-013-0018-x 23377696

[B7] Devlin J. Chang M.-W. Lee K. Toutanova K. (2018). Bert: Pre-training of deep bidirectional transformers for language understanding. https://arxiv.org/abs/1810.04805.

[B8] Freites-Martinez A. Santana N. Arias-Santiago S. Viera A. (2021). Using the Common Terminology Criteria for adverse events (CTCAE - version 5.0) to evaluate the severity of adverse events of anticancer therapies. Actas Dermosifiliogr. Engl. Ed. 112 (1), 90–92. 10.1016/j.ad.2019.05.009 32891586

[B9] Goldberger A. L. Amaral L. A. Glass L. Hausdorff J. M. Ivanov P. C. Mark R. G. (2000). PhysioBank, PhysioToolkit, and PhysioNet: Components of a new research resource for complex physiologic signals. Circulation 101 (23), E215–E220. 10.1161/01.cir.101.23.e215 10851218

[B10] González-Carvajal S. Garrido-Merchán E. C., 2020. Comparing BERT against traditional machine learning text classification.

[B11] Griffis D. Shivade C. Fosler-Lussier E. Lai A. M., 2016. A quantitative and qualitative evaluation of sentence boundary detection for the clinical domain. AMIA Jt Summits Transl Sci Proc, p.88–97. PMC5001746 27570656

[B12] Handler S. M. Altman R. L. Perera S. Hanlon J. T. Studenski S. A. Bost J. E. (2007). A systematic review of the performance characteristics of clinical event monitor signals used to detect adverse drug events in the hospital setting. J. Am. Med. Inf. Assoc. 14 (4), 451–458. 10.1197/jamia.M2369 PMC2244905 17460130

[B13] Hazell L. Shakir S. A. W. (2006). Under-reporting of adverse drug reactions: A systematic review. Drug Saf. 29 (5), 385–396. 10.2165/00002018-200629050-00003 16689555

[B14] Henry S. Buchan K. Filannino M. Stubbs A. Uzuner O. n2c2 Shared Task Participants 2020 2018 n2c2 shared task on adverse drug events and medication extraction in electronic health records. J. Am. Med. Inf. Assoc. 27 (1), 3–12. 10.1093/jamia/ocz166 PMC7489085 31584655

[B15] Hohl C. M. Small S. S. Peddie D. Badke K. Bailey C. Balka E. (2018). Why clinicians don't report adverse drug events: Qualitative study. JMIR Public Health Surveillance 4 (1), e21. 10.2196/publichealth.9282 29487041 PMC5849794

[B16] Huang K. Altosaar J. Ranganath R., 2019. ClinicalBERT: Modeling clinical notes and predicting hospital readmission.

[B17] Jagannatha A. Liu F. Liu W. Yu H. (2019). Overview of the first natural language processing challenge for extracting medication, indication, and adverse drug events from electronic health record notes (MADE 1.0). Drug Saf. 42 (1), 99–111. 10.1007/s40264-018-0762-z 30649735 PMC6860017

[B18] Joachims T. (1998). “Text categorization with support vector machines: Learning with many relevant features,” in Machine learning: ECML-98. Ecml 1998. Editors Nédellec C. Rouveirol C. (Berlin, Heidelberg: Springer), 1398. 10.1007/BFb0026683

[B19] Johnson A. E. W. Pollard T. J. Shen L. Lehman L. W. H. Feng M. Ghassemi M. 2016. MIMIC-III, a freely accessible critical care database. Sci. Data. 3, 160035 10.1038/sdata.2016.35 PMC4878278 27219127

[B20] Kaushal R. Jha A. K. Franz C. Glaser J. Shetty K. D. Jaggi T. (2006). Return on investment for a computerized physician order entry system. J. Am. Med. Inf. Assoc. 13 (3), 261–266. 10.1197/jamia.M1984 PMC1513660 16501178

[B21] Kim Y. (2014). “Convolutional neural networks for sentence classification,” in Proceedings of the 2014 conference on empirical methods in natural language processing (EMNLP) (Doha, Qatar: Association for Computational Linguistics), 1746–1751.

[B22] Lee J. Yoon W. Kim S. Kim D. Kim S. So C. H. (2020). BioBERT: A pre-trained biomedical language representation model for biomedical text mining. Bioinformatics 36 (4), 1234–1240. 10.1093/bioinformatics/btz682 31501885 PMC7703786

[B23] McHugh M. L. (2012). Interrater reliability: The kappa statistic. Biochem. Med. Zagreb. 22 (3), 276–282. 10.11613/bm.2012.031 23092060 PMC3900052

[B24] Mikolov T. Sutskever I. Chen K. Corrado G. S. Dean J. (2013). “Distributed representations of words and phrases and their compositionality,” in Advances in neural information processing systems, 26.

[B25] Minaee S. Kalchbrenner N. Cambria E. Nikzad N. Chenaghlu M. Gao J. (2022). Deep learning-based text classification: A comprehensive review. ACM Comput. Surv. (CSUR) 54 (3), 1–40. 10.1145/3439726

[B26] Nashed A. Zhang S. Chiang C. W. Hwang A. Riaz N. Presley C. J. (2021). Comparative assessment of manual chart review and ICD claims data in evaluating immunotherapy-related adverse events. Cancer Immunol. Immunother. 70 (10), 2761–2769. 10.1007/s00262-021-02880-0 33625533 PMC10992210

[B27] Neamatullah I. Douglass M. M. Lehman L.-w. H. Reisner A. Villarroel M. Long W. J. (2008). Automated de-identification of free-text medical records. BMC Med. Inf. Decis. Mak. 8, 32. 10.1186/1472-6947-8-32 PMC2526997 18652655

[B28] Pennington J. Socher R. Manning C. (2014). “GloVe: Global vectors for word representation,” in Proceedings of the 2014 conference on empirical methods in natural language processing (EMNLP) (Doha, Qatar.

[B29] Polepalli Ramesh B. Belknap S. M. Li Z. Frid N. West D. P. Yu H. (2014). Automatically recognizing medication and adverse event information from food and drug administration’s adverse event reporting system narratives. JMIR Med. Inf. 2 (1), e10. 10.2196/medinform.3022 PMC4288072 25600332

[B30] Poudel D. R. Acharya P. Ghimire S. Dhital R. Bharati R. (2017). Burden of hospitalizations related to adverse drug events in the USA: A retrospective analysis from large inpatient database. Pharmacoepidemiol. Drug Saf. 26 (6), 635–641. 10.1002/pds.4184 28233421

[B31] Qaiser S. Ali R. (2018). Text mining: Use of TF-IDF to examine the relevance of words to documents. Int. J. Comput. Appl. 181 (1), 25–29. 10.5120/ijca2018917395

[B32] Rommers M. K. Teepe-Twiss I. M. Guchelaar H-J. (2007). Preventing adverse drug events in hospital practice: An overview. Pharmacoepidemiol. Drug Saf. 16 (10), 1129–1135. 10.1002/pds.1440 17610221

[B33] Savova G. K. Masanz J. J. Ogren P. V. Zheng J. Sohn S. Kipper-Schuler K. C. (2010). Mayo clinical text analysis and knowledge extraction system (cTAKES): Architecture, component evaluation and applications. J. Am. Med. Inf. Assoc. 17 (5), 507–513. 10.1136/jamia.2009.001560 PMC2995668 20819853

[B34] Sherstinsky A., 2018. Fundamentals of recurrent neural network (RNN) and long short-term memory (LSTM) network.

[B35] Skentzos S. Shubina M. Plutzky J. Turchin A. (2011). “Structured vs. unstructured: factors affecting adverse drug reaction documentation in an EMR repository,” in AMIA annual symposium proceedings (American Medical Informatics Association), 2011, 1270. 22195188 PMC3243255

[B36] Stenetorp P. Pyysalo S. Topić G. Ohta T. Ananiadou S. Tsujii J. I. (2012). “BRAT: a web-based tool for NLP-assisted text annotation,” in Proceedings of the Demonstrations at the 13th Conference of the European Chapter of the Association for Computational Linguistics, 102–107.

[B37] Sun C. Qiu X. Xu Y. Huang X. (2019). “How to fine-tune BERT for text classification?,” in Chinese computational linguistics. CCL 2019. Editors Sun M. Huang X. Ji H. Liu Z. (Cham: Springer), 11856. 10.1007/978-3-030-32381-3_16

[B38] Vaswani A. Shazeer N. Parmar N. Uszkoreit J. Jones L. Gomez A. N. (2017). “Attention is all you need,” in Advances in neural information processing systems, 6000–6010.

[B39] Wang P. Bai G. R. Stolee K. T., 2019. Exploring regular expression evolution. Proceedings of the 2019 IEEE 26th International Conference on Software Analysis, Evolution and Reengineering (SANER), pp.502–513.

[B40] Watanabe J. H. McInnis T. Hirsch J. D. (2018). Cost of prescription drug–related morbidity and mortality. Ann. Pharmacother. 52 (9), 829–837. 29577766 10.1177/1060028018765159

[B41] Wei Q. Ji Z. Li Z. Du J. Wang J. Xu J. (2020). A study of deep learning approaches for medication and adverse drug event extraction from clinical text. J. Am. Med. Inf. Assoc. 27 (1), 13–21. 10.1093/jamia/ocz063 PMC6913210 31135882

[B42] Wishart D. S. Knox C. Guo A. C. Cheng D. Shrivastava S. Tzur D. (2008). DrugBank: A knowledgebase for drugs, drug actions and drug targets. Nucleic Acids Res. 36, D901–D906. 10.1093/nar/gkm958 18048412 PMC2238889

[B43] Xu G. Meng Y. Qiu X. Yu Z. Wu X. (2019). Sentiment analysis of comment texts based on BiLSTM. IEEE Access 7, 51522–51532. 10.1109/ACCESS.2019.2909919

