# Supplementary material for: Generalizability of machine learning methods in detecting adverse drug events from clinical narratives in electronic medical records
Source: Front Pharmacol. 2023 Jul 12;14:1218679. doi: 10.3389/fphar.2023.1218679 (PMC10368879; doi:10.3389/fphar.2023.1218679)
Supplement: Supplementary file 1 [file Table1.DOCX]

Supplementary Material

**Generalizability of machine learning methods in detecting adverse drug events from clinical narratives in electronic medical records**

Md Muntasir Zitu, BS^1^, Shijun Zhang, MS^1^, Dwight H. Owen, MD^2^, ChienWei Chiang, PhD^1^, Lang Li, PhD^1^

^1^Department of Biomedical Informatics, College of Medicine, The Ohio State University, Columbus, Ohio, USA; ^2^Department of Internal Medicine, College of Medicine, The Ohio State University, Columbus, Ohio, USA

Corresponding author: Lang Li, PhD, Professor and Chair, Department of Biomedical Informatics, College of Medicine, The Ohio State University, 250 Lincoln Tower, 1800 Cannon Drive, Columbus, Ohio 43210, USA (Lang.Li@osumc.edu) 614-685-4685.


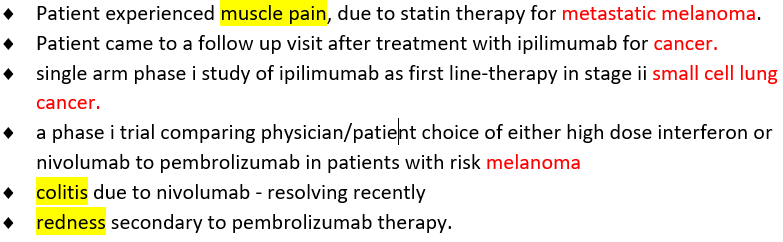


**Supplementary Figure 1: Annotation of adverse drug event (ADE).** Text in red highlights the reason for drug administration and should not be tagged as ADE, and text in yellow highlights true ADEs that should be tagged as ADE


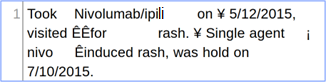


**Supplementary Figure 2: Input clinical note.** This figure is a chunk of an original clinical note.


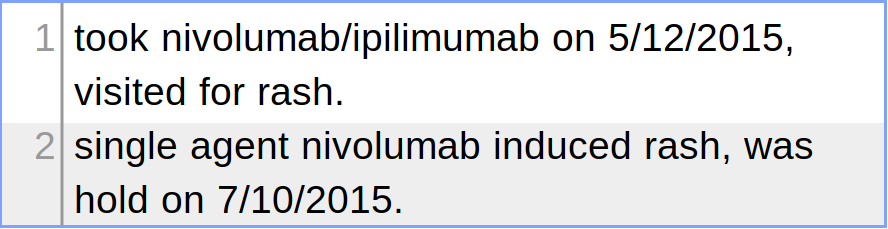


**Supplementary Figure 4: Sentence segmentation in cTAKES™** yielded two different sentences.


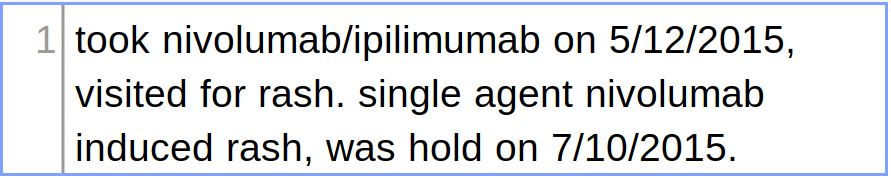


**Supplementary Figure 3: Cleaned data.** Appearance of original data after cleaning.


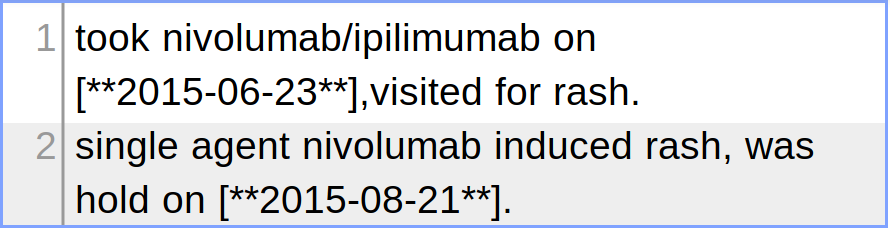


**Supplementary Figure 5: Auto-deidentification** in Physionet software yielded change in dates.


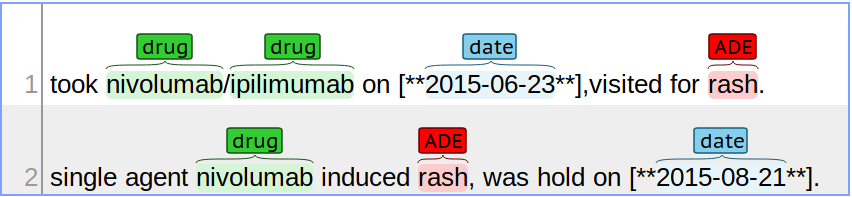


**Supplementary Figure 6: Automatic tagging** yields important entities, such as drug names, adverse drug events (ADEs), and dates.


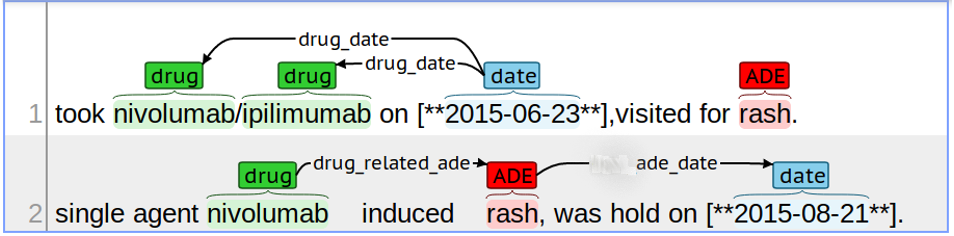


**Supplementary Figure 7 Manual annotation** by two annotators.

| Entity type | Annotation guideline |
| --- | --- |
| *drug* | This entity tag annotates drug name. Names of drugs include generic, trade, brand, and systematic names and abbreviations. DrugBank was used as a primary guideline for drug names. |
| *ADE* | This entity tag annotates adverse drug events. Mentions of adverse effects include signs, symptoms, disorders, diseases, deficiencies, acquired abnormalities, death, or organ damage that occurred strictly as a result of the drug. The *reason for drug administration* should not be tagged as ADE. Supplementary Figure 1 illustrates the difference between ADE and *reason for drug administration* with an example. Table 1 shows the lists of drugs and ADEs included in this study. |
| *date* | This entity tag type annotates the date a drug or an ADE was mentioned. To reduce the workload of annotators, all the dates were automatically tagged and de-identified with a shifted date by random number of days specific to a patient. |
| *causal_term* | This entity tag type annotates the action term indicating that the ADE was due to medical intervention related to a drug |
| *grade* | This entity tag type annotates the score of severity of the ADE based on the Common Terminology Criteria for Adverse Events (CTCAE) guideline (rating from 0 [no ADE] to 5 [severe ADE]). The intensity of severity (mild, moderate, and severe) should also be annotated. |

Supplementary Table 1: This table contains annotation guideline for entity tagging.

| Relation type | Annotation guideline |
| --- | --- |
| causal_term_drug | **This indicates relationship between a causal term and a drug associated with a drug-ADE relationship.** |
| drug_ade | **This indicates relationship between a drug and an ADE and** requires clear evidence that the ADE was induced by a drug. In the case of more than one drug or a drug combination related to an ADE, each is annotated separately with the ADE. |
| drug_date | **This indicates relationship between a drug and a date when the drug was taken.** |
| ade_date | **This indicates relationship between an ADE and the onset date of the ADE.** |
| ade_GRADE | **This indicates relationship between an ADE and the grade of this ADE.** |

Supplementary Table 2: This table contains annotation guideline for relation annotation.

| Relation count | | | Entity count | | |
| --- | --- | --- | --- | --- | --- |
| Relation type | Round 1 | Round 2 | Entity | Round 1 | Round 2 |
| Single-sentence  positive drug-adverse drug event (ADE) | 118 | 163 | Drug | 3822 | 4188 |
| Cross-sentence  positive drug-ADE | 24 | 27 | ADE | 8106 | 10094 |
| Single-sentence negative drug-ADE | 12 | 15 | Grade | 1331 | 492 |
| Cross-sentence negative drug-ADE | 4 | 39 | Causal Term | 282 | 139 |
| Grade-ADE relations | 343 | 466 |  |  |  |
| Drug-date relations | 1023 | 458 | Dates | 30716 | 26817 |
| ADE-date relations | 1321 | 287 | - | - | - |

Supplementary Table 3: Results of our two rounds of annotation.

| Model | Train | Test | Precision | Recall | F-score |
| --- | --- | --- | --- | --- | --- |
| SVM | ICI-OSU | n2c2 | 0.81 | 0.49 | 0.61 |
| SVM | n2c2 | ICI-OSU | 0.48 | 0.85 | 0.61 |
| BiLSTM | ICI-OSU | n2c2 | 0.76 | 0.68 | 0.72 |
| BiLSTM | n2c2 | ICI-OSU | 0.64 | 0.81 | 0.72 |
| CNN | ICI-OSU | n2c2 | 0.78 | 0.64 | 0.70 |
| CNN | n2c2 | ICI-OSU | 0.41 | 0.83 | 0.55 |
| BERT | ICI-OSU | n2c2 | 0.74 | 0.71 | 0.73 |
| BERT | n2c2 | ICI-OSU | 0.58 | 0.83 | 0.68 |
| ClinicalBERT | ICI-OSU | n2c2 | 0.76 | 0.80 | 0.78 |
| ClinicalBERT | n2c2 | ICI-OSU | 0.67 | 0.82 | 0.74 |

**Supplementary Table 4:** Train and test model on the data sets of different data sources. BERT, bidirectional encoder representations from transformers; BiLSTM, bidirectional long short-term memory; CNN, convolution neural network; ICI, immune checkpoint inhibitor; n2c2, 2018 National Clinical Challenge, Harvard University; OSU, The Ohio State University James Cancer Hospital; SVM, support-vector machine

| Model | Train |  | Test | Precision | Recall | F-score |
| --- | --- | --- | --- | --- | --- | --- |
| SVM | ICI-OSU |  | ICI-OSU | 0.80 | 0.75 | 0.77 |
| SVM | n2c2 |  | n2c2 | 0.75 | 0.91 | 0.83 |
| BiLSTM | ICI-OSU |  | ICI-OSU | 0.84 | 0.71 | 0.77 |
| BiLSTM | n2c2 |  | n2c2 | 0.76 | 0.93 | 0.84 |
| CNN | ICI-OSU |  | ISU-OSU | 0.82 | 0.78 | 0.80 |
| CNN | n2c2 |  | n2c2 | 0.72 | 0.92 | 0.81 |
| BERT | ICI-OSU |  | ICI-OSU | 0.80 | 0.69 | 0.74 |
| BERT | n2c2 |  | n2c2 | 0.81 | 0.85 | 0.83 |
| ClinicalBERT | ICI-OSU |  | ICI-OSU | 0.78 | 0.66 | 0.72 |
| ClinicalBERT | n2c2 |  | n2c2 | 0.84 | 0.90 | 0.87 |

**Supplementary Table 5:** Train and test model on the data set of same data source. BERT, bidirectional encoder representations from transformers; BiLSTM, bidirectional long short-term memory; CNN, convolution neural network; ICI, immune checkpoint inhibitor; n2c2, 2018 National Clinical Challenge, Harvard University; OSU, The Ohio State University James Cancer Hospital; SVM, support-vector machine
